# An Update on Imaging in Idiopathic Intracranial Hypertension

**DOI:** 10.3389/fneur.2020.00453

**Published:** 2020-06-10

**Authors:** David Moreno-Ajona, James Alexander McHugh, Jan Hoffmann

**Affiliations:** ^1^Basic and Clinical Neuroscience, Institute of Psychiatry, Psychology and Neuroscience, King's College London, London, United Kingdom; ^2^NIHR-Wellcome Trust King's Clinical Research Facility/SLaM Biomedical Research Centre, King's College Hospital, London, United Kingdom; ^3^Department of Ophthalmology, King's College Hospital NHS Trust, London, United Kingdom

**Keywords:** headache, idiopathic intracranial hypertension (iih), neuroimaging, optical coherence tomography, pain

## Abstract

Neuroimaging plays an essential role in the diagnostic workup of idiopathic intracranial hypertension with the aims to exclude secondary causes of elevated intracranial pressure and to identify imaging signs that are commonly observed in this disorder. As a valuable expansion of brain imaging, the imaging of the retina using optical coherence tomography has been of increasing value. In particular, this is the case with the latest devices that allow a more accurate distinction between a reduction in retinal nerve fiber layer thickness due to an improvement of papilledema or due to a worsening caused by optic nerve atrophy. Although optical coherence tomography does not yet replace the other elements of the diagnostic workup, it is likely to play an increasing role in diagnosis and follow-up of idiopathic intracranial hypertension. The review focuses on the main findings in neuroimaging, including structural and vascular alterations as well as on the relevance of optical coherence tomography.

## Introduction

Idiopathic intracranial hypertension (IIH) is defined as an elevation of intracranial pressure (ICP) in the absence of a brain lesion or any other secondary etiology (1). IIH generally affects obese young women of childbearing age. Given the relationship to obesity, the prevalence of IIH, which is currently estimated at 0.5–2.0 per 100,000 of the general population (2), is increasing along with the worldwide increasing incidence of obesity (3). The potential similarity of the clinical picture to primary headaches, in particular chronic migraine, probably results in IIH still being underdiagnosed (4).

In this article, we review our understanding of IIH with a special focus on the current imaging techniques and their utility in diagnosing and managing IIH.

### Clinical Picture

The clinical picture of IIH is dominated by headache and ophthalmic features resulting from the pressure-induced papilledema (1, 5). Headache is the most common clinical symptom of IIH and a key factor in the reduction of quality of life (4, 6). The headache can vary substantially in its clinical presentation, hence, the relatively unspecific definition in the diagnostic criteria of the International Headache Society. Frequently, the headache has a migraine phenotype, raising the question to what extent is the headache primarily driven by the elevated ICP or by a pressure-induced exacerbation of a pre-existing migraine. This uncertainty is fuelled further by the fact that most IIH patients do not experience a sustained improvement of their headache once ICP is normalized with an adequate treatment. Despite causing major morbidity in IIH and having an immense impact on patients' quality of life, no clinical trials exist that focus exclusively on the headache component of IIH (7).

The second cardinal feature of IIH is the papilledema caused by the elevation of ICP. In the majority of cases, papilledema is bilateral, but in up to 4% of cases, it can be asymmetrical. If IIH is untreated, the papilledema leads to numerous visual symptoms, including visual field defects, obscurations, and ultimately to the complete loss of eyesight resulting from an atrophy of the optic nerve. Due to the potential irreversibility of visual symptoms, a quick and accurate assessment is essential in the diagnostic workup of IIH.

### Pathophysiology

The pathophysiology remains unknown although our understanding has evolved significantly over the last decades. The first studies led to the belief that IIH may be due to increased CSF (8–10). This idea was refuted by Dandy and coworkers in the late 1930s as they saw no ventricular size alteration on ventriculography. The authors hypothesized IIH was related to an increased intracranial blood volume as a result of vasomotor control (11). Indeed, they suggested changes in the vascular bed would explain better the rapid changes in the intracranial pressure they observed. In the early 1950s, venography studies showed obstruction of the superior sagittal sinus (SSS) and dominant transverse sinus (12). Studies, including brain biopsy, in the late 1950s demonstrated intracellular and extracellular cerebral edema (13, 14). In the 1970s, after performing isotope cisternography and ventriculography, Johnston and coworkers hypothesized that a pressure increase within the SSS may lead to reduced CSF absorption (15). Raichle and coworkers, utilizing tracer techniques, showed a reduction in cerebral blood flow despite an increase in cerebral blood volume and pointed to an abnormality in the cerebral microvasculature (16). More recently, in 1995, with the use of cerebral venography and manometry, venous hypertension was shown in the SSS and the transverse sinus (17). In line with some studies performed in the 1930s and 1950s, a 3D volumetric MR imaging study showed normal ventricular volume in IIH. Nevertheless, the authors also observed increased extraventricular CSF volume (18). A phase-contrast MRI performed to measure the interaction between CSF and blood flows demonstrated the presence of a small phase shift of venous outflow leading to increased arteriovenous pulsatility, which ultimately would lead to an increase in CSF and (ICP) (19). The venous sinus stenosis hypothesis has led to venous sinus stenting as a therapy, the efficacy of which appears to be related to the pressure gradient prior to surgery (18).

Recently, the role obesity may play has also been addressed. A pathophysiological link is supported by reports of patients whose CSF opening pressure was normalized following bariatric surgery (20). Indeed, two cases showed reduction in venous sinus pressure as measured by intracranial venography following surgery (21). As a causative factor, recent evidence points to androgen excess, specifically testosterone, concentrations of which were found to be higher in both blood and CSF as compared to obese females with and without polycystic ovary syndrome (22).

### Structural MRI

Structural MRI is a key element in the diagnostic workup of IIH with the aim of ruling out a secondary cause of elevated ICP and to identify neuroimaging signs that are typically observed in IIH. One of the most suggestive neuroimaging abnormalities that is highly suggestive of IIH is the reduction of the midsagittal height of the pituitary gland (“empty sella”) (23). This is reflected in a significant reduction in its volume when performing an MR-based volumetric measurement (24). It is not entirely clear how a long-term increase of ICP causes the size reduction of the pituitary gland, but it is thought to be the result of a herniation of arachnocele through the diaphragma sellae (25). Interestingly, most abnormal morphometric neuroimaging findings do not improve after CSF pressure has been normalized and papilledema has resolved (26). However, healthy participants in research studies or patients who are scanned for a different reason may show an “empty sella.” Although, in the context of IIH, treatment should be based on the principle of treating clinical symptoms and not radiological signs, recent evidence suggests that a close follow-up of these patients may be recommendable (27).

Another typical neuroimaging finding in IIH is the distension of the optic nerve sheath (ONS) observed on T2-weighted MR-images (23, 24, 28). The distension of the ONS results from increased CSF pressure in the perioptic subarachnoid space. The adaptation of CSF pressure in the ONS to the ICP is not immediate due to the capillary CSF communication in the optic canal. For this reason, changes in the ONS are not seen in acute ICP changes (e.g., intracranial hemorrhage) (29) or within a few hours after normalization of ICP (30) although the exact time of the delay remains unknown. In contrast, although the ONS shows a macroscopic distention and the optic nerve may appear tortuous, the size and volume of the optic nerve remain unchanged (23, 24). However, when imaging is performed with diffusion tensor imaging (DTI) to analyze microstructural properties, changes in the optic nerve are identified (28, 30). These changes are in line with a microstructural tissue compression and are reversed after normalization of ICP (30). The fact that microstructural alterations within the optic nerve improve within 24 hours of lumbar puncture but the macroscopic size of the ONS does not highlights the delayed effect on the perioptic space after normalization of ICP and may suggest a higher sensitivity when imaging microscopic alterations using DTI compared to macroscopic changes in ONS using T2-weighted MRI. Nevertheless, the data from this study is based on a small number of patients and, therefore, requires a larger study to be confirmed (30). In line with microstructural imaging of the optic nerve, DTI of the optic disc shows abnormal values of fractional anisotropy in patients with IIH compared to healthy controls (31).

A posterior flattening of the optic globe is also commonly observed, but compared to the previously mentioned neuroimaging signs, it has an inferior sensitivity (23).

Finally, unilateral or bilateral transverse sinus stenoses (TSS) are commonly observed in IIH. Data on the prevalence of TSS in IIH vary substantially as MR-venography is frequently affected by imaging artifacts. It still remains controversial whether these are the cause or consequence of elevated ICP. However, increasing evidence suggest that TSS are secondary to increased ICP as they can resolve after normalization of ICP (32). The fact that bilateral stenting can resolve elevated ICP could be explained by a vicious cycle in which elevated ICP causes compression of the transverse sinuses, further aggravating the situation by obstructing venous outflow and thereby reducing the pressure gradient over the arachnoid villi (4, 33).

### Optical Coherence Tomography

Optical coherence tomography (OCT) uses a low-energy near-infrared laser beam that is projected onto the retina, and the light reflected from the retina interacts with a reference laser beam to create an interference pattern, which is analyzed to determine the reflectance of retinal tissue at different depths (34). Up to 100,000 points are scanned per second, creating exquisitely detailed profiles (axial resolution currently up to 3 μm) from which thickness maps of different retinal layers can be derived. Modern spectral-domain and swept-source OCTs use en-face laser ophthalmoscopic images of fundus vessels to ensure that follow-up scans in a given patient are exactly aligned with baseline scans, allowing tiny changes in retinal elevation and the thickness of individual retinal layers to be reliably measured.

#### OCT Measurement of Papilledema in IIH

OCT has a well-established role in assessing and monitoring papilledema (35–37). A number of different OCT scanning protocols are used to assess the optic disc in ophthalmology. The most widely used is a 3.4 mm line scan measuring retinal nerve fiber layer thickness (pRNFL). Papilledema causes thickening of the pRNFL, and greater thickness is associated with higher lumbar puncture opening pressure (35, 38, 39). In very early papilledema, retinal nerve fiber layer thickening may not extend far enough from the disc to be picked up by a pRNFL scan (40) although, in severe RNFL thickening, automated segmentation analysis is often unreliable, requiring manual correction to ensure valid longitudinal data (35).

**Figure 1:**
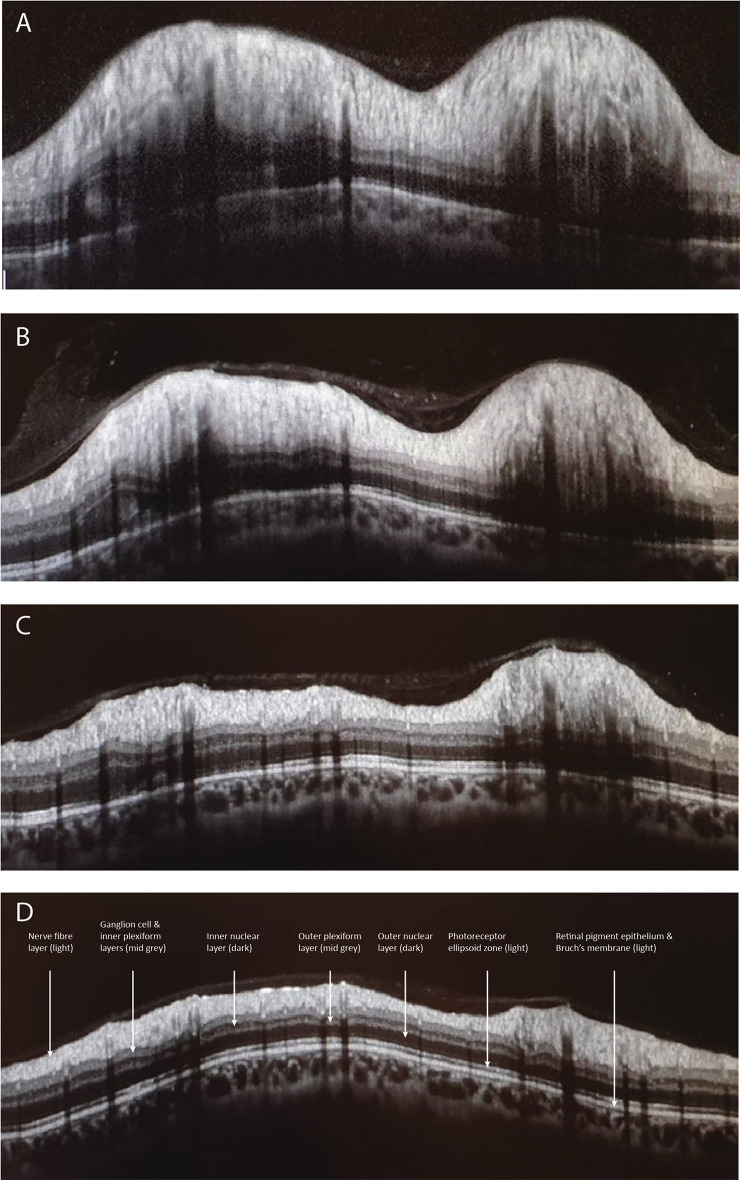
Depicts the peripapillary retinal nerve fiber thickness (pRNFL) scan performed with optical coherence tomography. Image **(A)** illustrates a pRNFL scan with severe disc swelling in IIH compared to day 5 **(B)**, day 30 **(C)**, and day 70 **(D)** after placing a ventriculoperitoneal shunt.

A variety of OCT scanning strategies have been described to quantify the elevation and volume of the disc itself in papilledema, which may offer some advantages over conventional pRNFL scans, especially in very early swelling (41–45). It has been shown that treatment of IIH with acetazolamide, successful weight loss, or ventriculo-peritoneal shunt causes corresponding improvement in OCT measures of disc height, volume, and pRNFL (43, 44, 46, 47) (Figure 1).

#### Deformation of the Peripapillary Retina

A number of methods have been described for measuring deformation of the layers deep into the neural retina (peri-papillary retinal pigment epithelium and Bowman's membrane) toward the vitreous, equivalent to inward deformation of the posterior sclera seen on MRI. The degree of deformation is related to lumbar puncture opening pressure and improves with ICP-lowering treatment (36, 41, 43, 48–50).

#### Diagnosis of Pseudopapilledema

OCT can readily distinguish tilted discs, the crowded hypermetropic discs, and buried disc drusen from true papilledema. The use of enhanced depth imaging allows OCT to image as deep as the lamina cribrosa of the sclera to detect even very small drusen (51–53).

#### OCT Macular Ganglion Cell Layer Imaging in IIH

**Figure 2:**
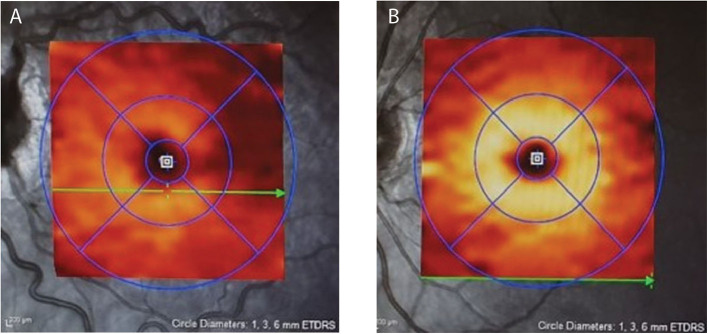
Depicts the moderate macular ganglion cell layer (mGCL) thinning in IIH **(A)** vs. a healthy control **(B)**. Note on image A the dilated, tortuous veins resulting from papilledema.

A significant challenge in monitoring IIH-related papilledema is to determine whether a reduction in the degree of disc or pRNFL swelling is due to improvement of edema due to falling ICP from successful treatment or, conversely, to the loss of RNFL fibers as optic atrophy develops. Macular OCT imaging is extremely helpful in this situation. The macular ganglion cell layer (mGCL), which contains the cell bodies of axons of the optic nerve, does not swell in papilledema. Disc damage due to papilledema causes early thinning of the mGCL before frank thinning of the pRNFL develops (47, 54) (Figure 2). Conversely, finding that a patient with chronic papilledema despite medical therapy has no thinning (or no progression of thinning) of the mGCL offers reassurance that the optic nerve is not losing axons at an abnormal rate.

#### Other Applications of OCT Systems in IIH

Various other OCT features may have value in diagnosing or monitoring IIH. These include imaging retinal and choroidal folds due to papilledema, of which some subtypes may improve with treatment (55, 56); OCT imaging of venular diameter, which increases in papilledema and decreases when elevated ICP is reduced (57); and OCT angiographic imaging of peripapillary capillaries, which have increased diameter and tortuosity in papilledema (58).

In patients suspected of having IIH without papilledema or in whom established optic atrophy prevents disc swelling, OCT systems can be used to obtain motion-stabilized laser ophthalmoscopic videos, which are extremely sensitive in detecting spontaneous retinal venous pulsation (SVP) (59). The presence of SVP signifies a healthy pressure gradient between the eye and the retrobulbar perineural CSF, and videography using OCT systems has shown that SVP reliably disappears when ICP becomes moderately elevated (60).

## Conclusions

Neuroimaging in IIH has substantially improved diagnostic accuracy in IIH. Although it is unlikely that it will replace diagnostic lumbar puncture, it is feasible that, in a few years, improved MR imaging, including microstructural imaging as well as the rapidly improving quality of OCT imaging of papilledema, may offer a possibility to reduce the number of lumbar punctures for diagnostic follow-up as they could provide reliable markers that could be used in indirectly assessing ICP (32, 61).

## Author Contributions

DM-A, JM, and JH performed the literature review and drafted the manuscript.

## Conflict of Interest

JH is consulting for and/or serves on advisory boards of Allergan, Autonomic Technologies, Inc. (ATI), Chordate Medical AB, Eli Lilly, Hormosan Pharma, Novartis and Teva. He has received honoraria for speaking from Allergan, Chordate Medical AB, Novartis and Teva. He received personal fees for Medico-Legal Work as well as from Sage Publishing, Springer Healthcare and Quintessence Publishing. The remaining authors declare that the research was conducted in the absence of any commercial or financial relationships that could be construed as a potential conflict of interest.
